# A novel *SMAD6* variant in a patient with severely calcified bicuspid aortic valve and thoracic aortic aneurysm

**DOI:** 10.1002/mgg3.620

**Published:** 2019-03-08

**Authors:** Jong Eun Park, Jin Seok Park, Shin Yi Jang, Seok Hee Park, Jong‐Won Kim, Chang‐Seok Ki, Duk‐Kyung Kim

**Affiliations:** ^1^ Department of Laboratory Medicine and Genetics Samsung Medical Center, Sungkyunkwan University School of Medicine Seoul Korea; ^2^ Department of Biological Sciences Sungkyunkwan University Suwon Korea; ^3^ Division of Cardiology, Department of Medicine Heart Vascular Stroke Institute, Samsung Medical Center, Sungkyunkwan University School of Medicine Seoul Korea; ^4^ Green Cross Genome Yongin Korea

**Keywords:** bicuspid aortic valve, *SMAD6*, whole exome sequencing

## Abstract

**Background:**

Bicuspid aortic valve (BAV) is the most common congenital heart defect with a prevalence of 1%–2% in the general population. *NOTCH1*, *SMAD6*, and *GATA5 *are associated with BAV in humans, but few cases have been reported that did not involve *NOTCH1*. Here, we identified novel in‐frame variants in *SMAD6* (c.1168_1173dup; p.Gly390_Ile391dup) in a BAV patient, who presented with dilatation of the ascending aorta and severe calcification of the aortic valve.

**Methods:**

Twenty BAV associated genes were screened by exome sequencing. Functional effects of *SMAD6* variant were investigated using bone morphogenetic protein (BMP) signaling assays through in vitro functional study.

**Results:**

Exome sequencing revealed he had novel in‐frame variants in the *SMAD6* gene (c.1168_1173dup; p.Gly390_Ile391dup). SMAD6 is known to be an inhibitory protein in the BMP signaling pathway. In vitro functional study of the p.Gly390_Ile391dup variant revealed impaired inhibition of BMP signaling and BMP‐induced alkaline phosphatase activity.

**Conclusion:**

In conclusion, we identified a novel *SMAD6* variant causing a severely calcified BAV and TAA, which contributes to our understanding of the clinical and genetic background of *SMAD6*‐related BAV.

## 1 INTRODUCTION

Bicuspid aortic valve (BAV) is the most common congenital heart defect with a prevalence of 1%–2% in the general population (Michelena et al., 2014). BAV occurs more frequently in male Caucasians than females and non‐Caucasians. BAV is often associated with the occurrence of other cardiac manifestations such as a thoracic aortic aneurysm (TAA) or coarctation of the aorta (CoA) (Prakash et al., 2014). The risk of aortic dissection in BAV is 8.4 times higher than in the general population (Michelena et al., 2011).

Autosomal‐dominant inheritance may explain some but not all BAV families, suggesting an incomplete penetrance or complicated transmission trait (Prakash et al., 2014). To date, *NOTCH1*, *SMAD6*, and *GATA5 *have been known to be associated with BAV, but only a few human cases have been reported except for those involving *NOTCH1 *(Kassab et al., 2016; Tan et al., 2012). Genetic causes remain unknown in most of the BAV patients.

Recent study using variant burden analysis had suggested that *SMAD6* could be a significant contributor of BAV associated TAA in patients (Gillis et al., 2017). SMAD6, which is an inhibitory protein in the bone morphogenetic protein (BMP) signaling pathway, is highly expressed in heart and blood vessels (Galvin et al., 2000). This negative regulation of SMAD6 inhibited BMP signaling is important during heart development and homeostasis of the cardiovascular system.

Here, we identified novel in‐frame variants in *SMAD6* (c.1168_1173dup; p.Gly390_Ile391dup) in a BAV patient, who presented with dilatation of the ascending aorta and severe calcification of the aortic valve. Through a functional study, we found that the *SMAD6* in‐frame variant is related to severe calcification in the aortic valve.

## 2 MATERIALS AND METHODS

### 2.1 Patient and clinical investigation

Patients were evaluated by clinical examination, computed tomography (CT) angiography of the aorta, and echocardiography. The study was approved by the Institutional Review Board (SMC 2016‐11‐039) and the patient and his family provided written informed consent for clinical and genetic investigations.

### 2.2 Exome sequencing and data analysis

Genomic DNA was extracted from peripheral blood leukocytes using the Wizard Genomic DNA Purification Kit following the manufacturer's instructions (Promega, Madison, WI). SureSelect Human All Exon V6 (Agilent Technologies, Santa Clara, CA) was used for library preparation; sequencing was performed using the Illumina HiSeq2500 platform (Illumina Inc., San Diego, CA), generating 2 × 150‐bp paired‐end reads. Alignment of sequence reads was performed against the Human Reference Genome build GRCh37 using BWA 0.7.12; duplicated reads were marked with Picard Tools 1.130; local alignment, base quality recalibration, and variant calling were performed with the Genome Analysis Tool Kit v3.4.0; and annotation and variant effect prediction were performed with SnpEff v4.1g. The called variants were filtered and prioritized using a four‐step strategy. Initially, we screened 20 genes known to be associated with BAV (*NOTCH1, MAT2A, TGFBR2, ARHGAP31, MATR3, NKX2.5, MAML1, JARID2, ENG, ACTA2, MYH6, MYH7, FBN1, SMAD6, AXIN1, PDIA2, KCNJ2, SMARCA4, JAG1, *and* GATA5*) (Debiec, Sall, Samani, & Bolger, 2017). Next step, we removed variants below 10× coverage. The minor allele frequency (MAF) threshold was carefully chosen based on BAV prevalence and variants with an MAF ≥5% in the Exome Aggregation Consortium (ExAC) database (http://exac.broadinstitute.org/), the Genome Aggregation Database (gnomAD) (http://gnomad.broadinstitute.org/), or the Korean Reference Genome Database (KRGDB) (http://152.99.75.168/KRGDB/) were removed. The fourth step was to include variants that are predicted to have a high impact on protein function, including missense, nonsense, frameshifts, in‐frame insertions/deletions variants, or changes affecting the consensus splice site sequences. The candidate variant was confirmed using standard PCR and Sanger sequencing methods (primer sequences available upon request). Candidate variants were classified according to the standards and guidelines of the American College of Medical Genetics and Genomics (ACMG) and the Association for Molecular Pathology (AMP) (Richards et al., 2015). These guidelines recommend that the variants are classified into five categories: pathogenic variant, likely pathogenic variant, variant of uncertain significance, likely benign variant, and benign variant.

### 2.3 In vitro functional study

#### 2.3.1 Cell culture and transfection

Mouse myoblast C2C12 cells (American Type Culture Collection, LGC Standards, Teddington, UK) were maintained in Dulbecco's modified Eagle's medium in 10% fetal bovine serum with 50 units/ml penicillin and 50 μl/ml streptomycin (GIBCO‐BRL, Grand Island, NY) at 37°C in a 5% CO_2_‐humidified atmosphere. Cells were transfected using Lipofectamine 2000 transfection reagent (Invitrogen, Carlsbad, CA) according to the manufacturer's recommendations.

#### 2.3.2 Luciferase assay

Dual‐luciferase assays were performed using previously validated luciferase transcriptional reporter constructs containing BMP/SMAD‐responsive elements (BRE–luc) (Korchynskyi & ten Dijke, 2002). After C2C12 cells were seeded in 12‐well plates for 24 hr, plasmids encoding BRE–luc, wild‐type (wt) SMAD6, mutant SMAD6 constructs, or empty vector pcDNA3.1 (Invitrogen) were transfected according to the indicated combinations and subsequently treated with BMP2. A pGL4 vector (in which Renilla luciferase is driven by a thymidine kinase promoter) was cotransfected in all samples as a control for transfection efficiency. Cells were incubated for 24 hr after transfection and luciferase activities were measured in lysates using the Dual‐Luciferase Reporter Assay System (Promega) following the manufacturer's protocol. Data were normalized using Renilla luciferase activity. Three independent experiments were performed (each in triplicate).

#### 2.3.3 Immunoblotting

The immunoblot analysis was performed as described previously (Lee et al., 2017). The antibodies used for immunoblotting were as follows. Mouse anti‐Flag (F3165) and mouse anti‐β‐actin (A5316) were obtained from Sigma‐Aldrich (St. Louis, MO). Rabbit anti‐p‐Smad1/5/8 was purchased from Cell Signaling Technology (Danvers, MA). Mouse anti‐Smad4 (sc‐7966) and rabbit anti‐Smad1/5/8 (sc‐6031) were purchased from Santa Cruz Biotechnology (Dallas, TX).

#### 2.3.4 Alkaline phosphatase assay

Alkaline phosphatase (ALP) activity was measured via ALP staining in C2C12 cells transfected with SMAD6 variants. Briefly, cells were transfected with either wt SMAD6, mutant SMAD6 constructs, or empty vector pcDNA3.1 and subsequently treated with BMP2. These cells were further cultured for 2 days with one change of medium. Differentiated osteoblasts were equilibrated with ALP buffer, this was followed by the application of 0.4 mg/ml and 0.2 mg/ml of the staining solutions, nitro blue tetrazolium (NBT, GeorgiaChem, Norcross, GA) and 5‐bromo‐4‐chloro‐3‐indolyl‐phosphate (BCIP, Georgiachem), respectively. The cells were incubated for 15 min at room temperature for ALP staining. The reaction was stopped by adding PBS containing 5 mM EDTA.

## 3 RESULTS

### 3.1 Clinical features

**Figure 1:**
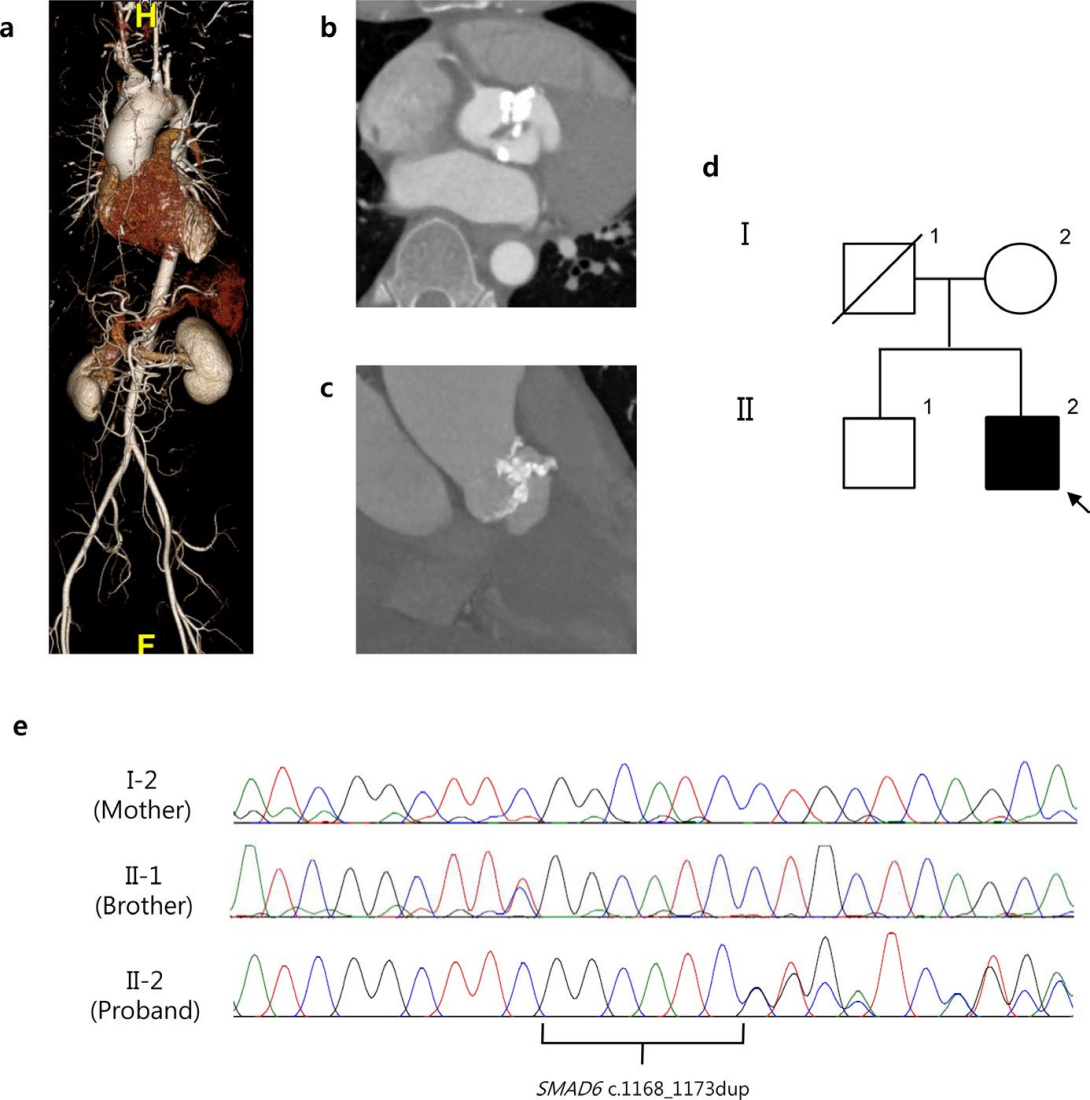
Computed tomography (CT) angiography of the aorta shows ascending aortic aneurysm (a) and severe calcification of the aortic valve (b, c). Pedigree of the family with bicuspid aortic valve (BAV) (d) and results of the SMAD6 c.1168_1173dup (p.Gly390_Ile391dup) mutation analysis for the proband and family members (e)

A 42‐year‐old man visited Samsung Medical Center due to an ascending aortic dilatation and echocardiography revealed BAV with severe calcification of the aortic valve. CT angiography of the aorta showed significant dilatation of the ascending aorta (diameter: 5.5 cm) and dense calcification in the aortic valve (Figure 1). Echocardiographic measurements of the aortic root size at the aortic valve annulus, sinus of Valsalva, and the sinotubular junction (STJ) were 2.5 cm, 3.1 cm, and 3.3 cm, respectively. Father of the proband died due to bladder cancer at the age of 69 without cardiovascular disease. No other family member had a history of cardiovascular disease.

### 3.2 Genetic analysis

We screened 20 genes known to be associated with BAV (Debiec et al., 2017). In *SMAD6*, a novel in‐frame variant (c.1168_1173dup; p.Gly390_Ile391dup) was identified. This p.Gly390_Ile391dup variant was not found in any of the public population databases such as 1,000 Genomes, the Genome Aggregation Database (gnomAD), and the Korean Reference Genome Database (KRGDB). The p.Gly390_Ile391dup was located in the MH2 domain of *SMAD6*, the function of which is critical for the protein's interaction with other members of the BMP signaling pathway. The p.Gly390_Ile391dup was not observed in the patient's mother and brother, who do not have BAV or TAA (Figure 1).

### 3.3 In vitro functional study

**Figure 2:**
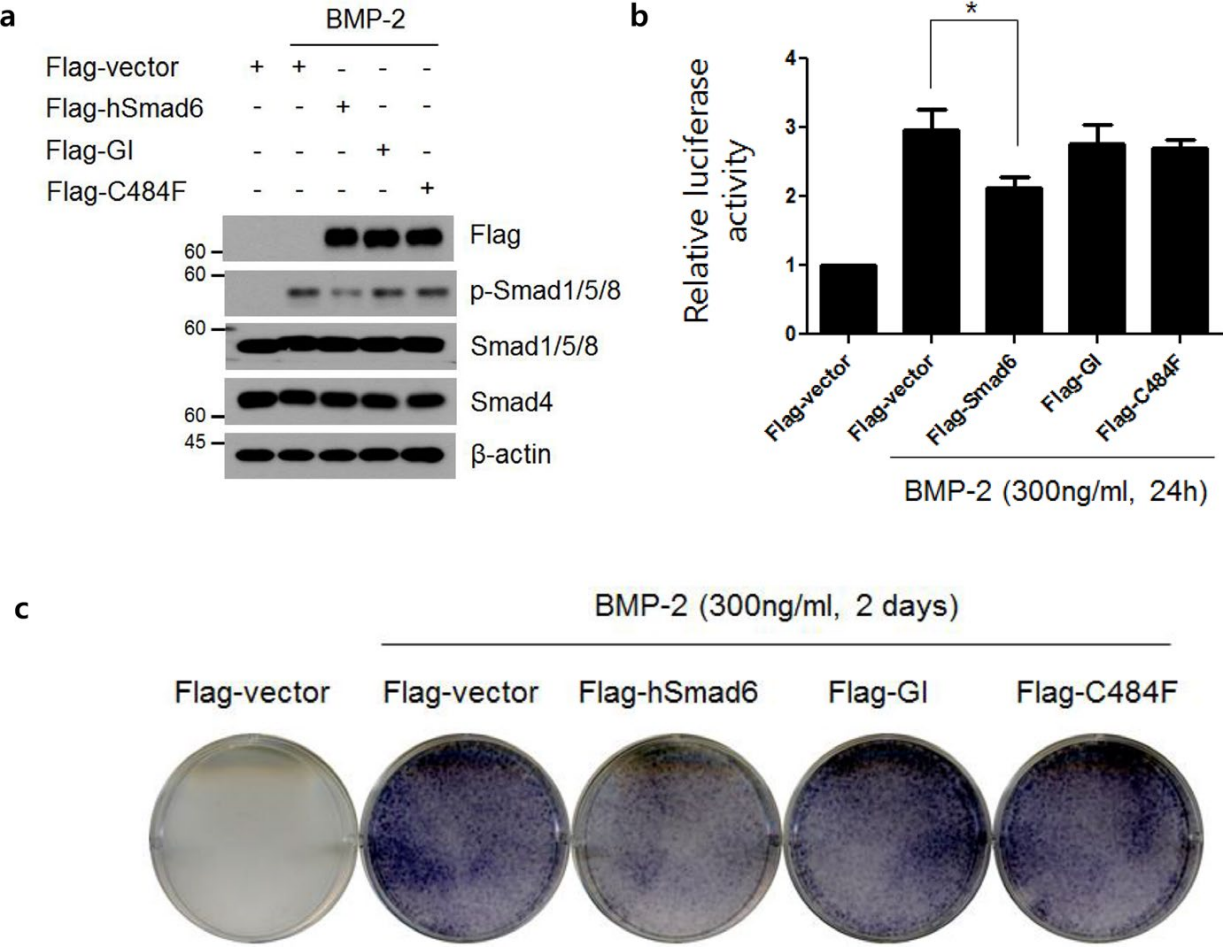
Inhibitory effects of *SMAD6* mutants in the BMP signaling pathway. (a) Plasmids encoding Flag‐tagged Smad6 (Flag‐hSmad6), Flag‐tagged SMAD6 p.Gly390_Ile391dup (Flag‐GI), and Flag‐tagged C484F (Flag‐C484F) were transfected into C2C12 cells. Cells were treated with BMP‐2 (300 ng/ml) for 30 min and were analyzed by immunoblotting (IB) with the antibodies indicated. (b) A plasmid encoding Flag‐hSmad6, Flag‐GI, and Flag‐C484F were cotransfected with the BRE–luc reporter into C2C12 cells under BMP‐2 (300 ng/ml) treatment for 24 hr. Luciferase activity was measured and normalized to the expression of *Renilla* luciferase. The data were statistically analyzed by one‐way ANOVA followed by Tukey's multiple comparison test (*p < 0.05, *n = *3). Bars represent the mean ± *SD*. (c) Alkaline phosphatase (ALP) stained C2C12 cells were transfected with Flag‐hSmad6, Flag‐GI, and Flag‐C484F followed by BMP‐2 treatment for 2 days

To gain further insight into the relationships between BMP signaling and the SMAD6 p.Gly390_Ile391dup variant, we generated wild‐type (wt) Smad6 and two different Smad6 mutants: Flag‐tagged Smad6 (Flag‐hSmad6), Flag‐tagged SMAD6 p.Gly390_Ile391dup (Flag‐GI), and Flag‐tagged C484F (Flag‐C484F). The p.C485F mutant, which was reported by Tan et al. (Tan et al., 2012) was used as a positive control. After plasmids encoding Flag‐hSmad6 and Smad6 mutants were transfected into C2C12 cells, the inhibitory effects of the *SMAD6* mutants on the BMP signaling pathway were assessed by immunoblotting, BRE–luc reporter assay, and ALP activity. Flag‐GI and Flag‐C484F showed impaired phosphorylation of SMAD1/5/8 under BMP‐2 treatment, which implies that the mutant SMAD6 inhibited BMP signaling less efficiently than Flag‐hSmad6 (Figure 2a). Luciferase activity of the BRE–luc reporter also showed a corresponding result. Flag‐hSmad6 inhibited luciferase activity, but Flag‐GI and Flag‐C484F could not (Figure 2b). Osteogenic potential was assessed by ALP staining, a commonly used early marker of osteoblast differentiation. Flag‐hSmad6 inhibited osteoblast differentiation. In contrast, Flag‐GI and Flag‐C484F showed impaired inhibition, suggesting that the mutant protein had less efficacy in preventing tissue calcification (Figure 2c).

## 4 DISCUSSION

In this study, we first confirmed and described that an in‐frame duplication variant affects the BMP signaling pathway and is associated with BAV and severe calcification in a TAA patient. To date, only 13 cases have been reported in two studies with human *SMAD6*‐related BAV patients. A summary of the clinical and mutational features in all cases are described in Table 1. Most cases of *SMAD6*‐related BAV patients have other cardiovascular anomalies such as TAA or CoA. Including the mutation in this study, 57.1% (8/14) variants were missense, 57.1% (8/14) were nonsense or frameshift variants, and 14.3% (2/14) were in‐frame variants. Two missense variants (p.Cys484Phe and p.Pro415Leu) were previously demonstrated to be clearly deleterious to function in a BMP signaling assay (Tan et al., 2012).

**Table 1:** Clinical and genetic characteristics of *SMAD6*‐related bicuspid aortic valve in the literature

Case No.	Sex	Age (years)	BAV subtype	Other cardiac manifestation	Nucleotide change	Protein change	Domain	gnomAD MAF	SIFT	Polyphen‐2	Ref
1	M	30	Unknown	AS, CoA, calcification	c.1451G>T	p.(Cys484Phe)	MH2	5.E‐06	Deleterious	Probably Damaging	Tan et al. (2012)
2	NA	18m	Unknown	moderate AS	c.1244C>T	p.(Pro415Leu)	MH2	absent	Deleterious	Probably Damaging	Tan et al. (2012)
3	M	NA	Unknown	TAA	c.74_79del	p.(Ser27_Gly28del)	other	4.E‐04	NA	NA	Gillis et al. (2017)
4	M	NA	Unknown	TAA	c.465_471del	p.(Gly156Valfs*23)	MH1	absent	NA	NA	Gillis et al. (2017)
5	M	NA	Unknown	TAA	c.715G>A	p.(Val239Met)	MH1	6.E‐05	Tolerated	Probably Damaging	Gillis et al. (2017)
6	M	NA	LR	TAA	c.726del	p.(Lys242Asnfs*297)	MH1	absent	NA	NA	Gillis et al. (2017)
7	F	NA	Unknown	TAA	c.770C>T	p.(Pro257Leu)	MH1	absent	Tolerated	Possibly damaging	Gillis et al. (2017)
8	M	NA	Unknown	TAA	c.812G>A	p.(Gly271Glu)	MH1	absent	Tolerated	Possibly damaging	Gillis et al. (2017)
9	F	NA	LR	TAA	c.837C>A	p.(Tyr279*)	other	absent	NA	NA	Gillis et al. (2017)
10	F	NA	LR	TAA, CoA	c.864C>G	p.(Tyr288*)	other	absent	NA	NA	Gillis et al. (2017)
11	M	NA	RN	TAA	c.1216G>T	p.(Gly406Cys)	MH2	absent	Deleterious	Probably Damaging	Gillis et al. (2017)
12	F	NA	RN	TAA	c.1224C>G	p.(His408Gln)	MH2	absent	Tolerated	Probably Damaging	Gillis et al. (2017)
13	M	NA	Unknown	TAA	c.1328G>A	p.(Arg443His)	MH2	4.E‐06	Deleterious	Benign	Gillis et al. (2017)
14	M	42	LA	AS, TAA, valve calcification	c.1168_1173dup	p.(Gly390_Ile391dup)	MH2	absent	NA	NA	This study

Note

AS: aortic stenosis; BAV: bicuspid aortic valve; CoA: coarctation of the aorta; gnomAD: Genome Aggregation Database; LA: lateral; LR: left‐right; NA: not applicable; MAF: minor allele frequency; RN: right‐non‐coronary; TAA: thoracic aortic aneurysm.

SMAD6 encodes an inhibitory SMAD protein which prevents phosphorylation of SMAD1/5/8 or competitive interaction for SMAD4 (Hata, Lagna, Massague, & Hemmati‐Brivanlou, 1998). As a result, SMAD6 negatively regulates the BMP signaling pathway. SMAD6 has two functionally important MH1 and MH2 domains. The MH1 domain binds to DNA (Bai & Cao, 2002), while the MH2 domain interacts with transforming growth factor β (TGF‐β) and the BMP signaling pathway (Bai & Cao, 2002; Lin et al., 2003). Among the human *SMAD6 *variants in BAV, six missense or in‐frame variants were located in the MH2 domain of SMAD6 (Table 1).

A patient with p.Gly390_Ile391dup had severe calcification in the aortic valve. According to Ankeny et al., reduced SMAD6 expression was associated with calcification of the aortic valve (Korchynskyi & ten Dijke, 2002). p.Gly390_Ile391dup showed impaired inhibition of osteoblast differentiation. This could confirm that severe calcification of the aortic valve was related to the *SMAD6* variant in this patient.

Many BAV patients have TAA, which was attributed to the hemodynamic consequence of BAV or the effect of genetic variation. Yassine et al. proposed that aortic root dilation below the STJ was strongly associated with genetic etiology and ascending aortic dilatation above the STJ was associated with hemodynamic influence (Yassine, Shahram, & Body, 2017). In this study, a patient with p.Gly390_Ile391dup had ascending aortic dilatation above the STJ, which might be attributed to a hemodynamic stress such as high‐velocity and turbulent flow induced by a severely stenotic BAV.

*SMAD6* is associated with not only BAV but also susceptibility of craniosynostosis (Timberlake et al., 2016). However, patient with p.Gly390_Ile391dup had no other craniofacial abnormalities.

Finally, this *SMAD6* p.Gly390_Ile391dup variant could be considered a “likely pathogenic” variant according to the 2015 ACMG‐AMP guidelines because (a) it is well‐established in functional studies in vitro that the variant has a damaging effect on the gene, (b) the variant is absent in large population databases, and (c) the protein length changes as a result of in‐frame deletions/insertions in a nonrepeat region.

In conclusion, we have identified a novel “likely pathogenic” variant (c.1168_1173dup; p.Gly390_Ile391dup) in the *SMAD6* gene. This report will contribute to a better understanding of the genetic background in patients with *SMAD6*‐related BAV.

## CONFLICT OF INTEREST

The authors have no conflicts of interest to report.
